# Interpreting Diastolic Dynamics and Evaluation through Echocardiography

**DOI:** 10.3390/life14091156

**Published:** 2024-09-12

**Authors:** Xiaoxiao Zhang, Ke Li, Cristiano Cardoso, Angel Moctezuma-Ramirez, Abdelmotagaly Elgalad

**Affiliations:** 1 Center for Preclinical Surgical and Interventional Research, The Texas Heart Institute, Houston, TX 77030, USA; xzhang@texasheart.org (X.Z.); ccardoso@texasheart.org (C.C.); amoctezuma@texasheart.org (A.M.-R.); 2 Internal Medicine, School of Medicine, University of Nevada, Reno, NV 89509, USA; keli@med.unr.edu

**Keywords:** diastolic dynamics, echocardiography evaluation, diastolic dysfunction, stress echocardiography, strain imaging, integrating artificial intelligence

## Abstract

In patients with heart failure, evaluating left ventricular (LV) diastolic function is vital, offering crucial insights into hemodynamic impact and prognostic accuracy. Echocardiography remains the primary imaging modality for diastolic function assessment, and using it effectively requires a profound understanding of the underlying pathology. This review covers four main topics: first, the fundamental driving forces behind each phase of normal diastolic dynamics, along with the physiological basis of two widely used echocardiographic assessment parameters, E/e’ and mitral annulus early diastolic velocity (e’); second, the intricate functional relationship between the left atrium and LV in patients with varying degrees of LV diastolic dysfunction (LVDD); third, the role of stress echocardiography in diagnosing LVDD and the significance of echocardiographic parameter changes; and fourth, the clinical utility of evaluating diastolic function from echocardiography images across diverse cardiovascular care areas.

## 1. Introduction

In heart failure (HF) patients, assessing left ventricular (LV) diastolic function is pivotal for understanding HF hemodynamics and improving prognostic accuracy [1,2]. Echocardiography is the primary imaging modality for this assessment, often providing comprehensive data [3]. Understanding the pathology revealed by 2D and Doppler imaging and stress echocardiography (SE) is crucial for accurate evaluation. Echocardiographers must grasp the physiological rationale for each parameter these techniques measure, the factors affecting reliability, and the technical acquisition and analysis of data.

## 2. Normal LV Filling Dynamics

**Figure 1:**
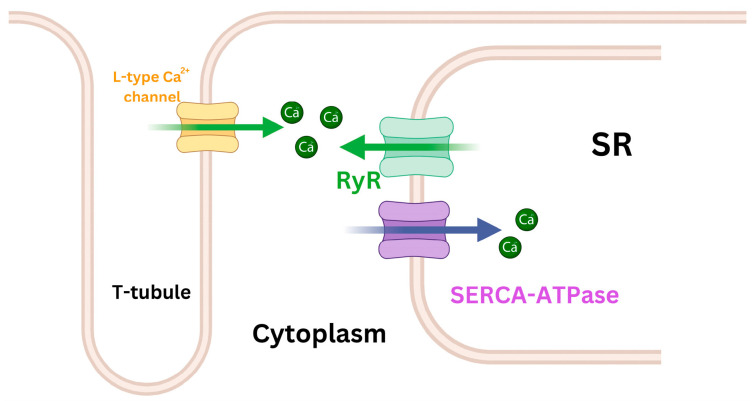
Simplified schematic diagram of Ca^2+^ regulation channels during the myocardial cell contraction and relaxation phases. The sarcoendoplasmic reticulum calcium ATPase (SERCA-ATPase) pump is vital for absorbing Ca^2+^ and storing it in the sarcoplasmic reticulum (SR). This absorption reduces the Ca^2+^ concentration in cytoplasm, contributing to the initiation of the myocyte’s relaxation phase. Importantly, SERCA-ATPase remains active throughout the relaxation process. In parallel, ryanodine receptors (RyR), stimulated by external Ca^2+^ from L-type Ca^2+^ channels, release stored Ca^2+^ from the SR. This increases the concentration of Ca^2+^ in the cytoplasm, ultimately triggering myocyte contraction.

During LV ejection, energy is stored as myocytes undergo compression, while the myocardial wall’s elastic components are also compressed and twisted [4]. Subsequently, at the end of systole, calcium ions are actively reabsorbed into the sarcoplasmic reticulum (SR) (Figure 1). This process, known as uncoupling, facilitates the detachment and repositioning of actin and myosin filament cross-bridges to their original configuration, thereby enabling muscle relaxation. This phase, known as “active relaxation”, involves adenosine triphosphate (ATP) consumption and does not occur instantaneously. Concurrently, during LV diastole, restoring forces further facilitate this relaxation phase. When contracted myocardium relaxes and untwists, stored energy is released as the elastic elements recoil [5], serving as the driving force in the early diastolic phase to aid myocardial fibers in extending from their minimum length during the contraction phase to their original length. The elastic recoil of the base and apex from their previous systolic positions, resulting in the release of the contracted LV myocardium along the longitudinal axis, is referred to in the literature by various names, including the LV untwisting motion [6], restoring forces [7], and elastic recoil [8]. Elastic recoil is passive relaxation, during which the myocardium spontaneously relaxes without consuming energy.

**Figure 2:**
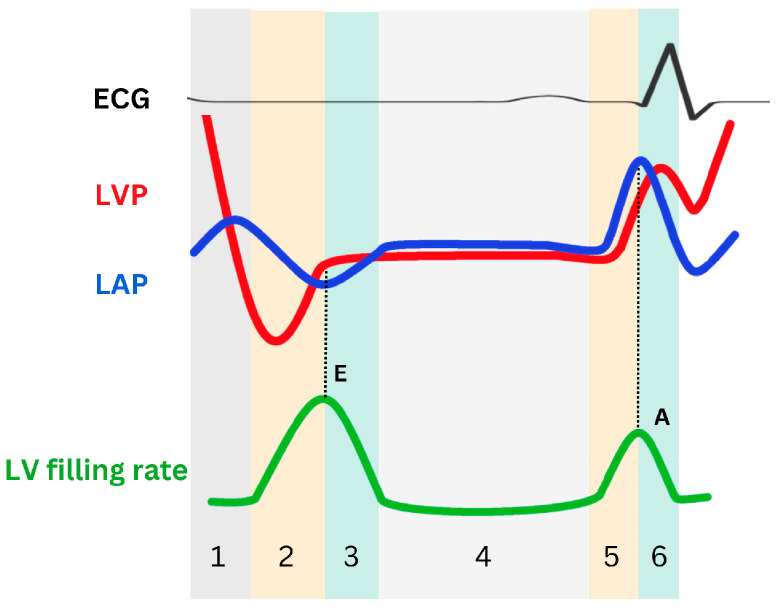
Schematic representation of left ventricular pressure (LVP), left atrial pressure (LAP), and LV filling rate during the relaxation phase. The cardiac cycle consists of six phases: isovolumetric relaxation (phase 1), LV active relaxation (early rapid filling in phase 2 and inflow deceleration in phase 3), diastasis (phase 4), and LA contraction (the A wave upstroke in phase 5 and the A wave downstroke in phase 6). The initial pressure crossover marks the conclusion of isovolumic relaxation and the commencement of early rapid filling. which begins with the opening of the mitral valve. During this phase, LAP surpasses LVP, hastening mitral flow, and peak mitral E aligns with the second crossover. Subsequently, LVP surpasses LAP, slowing mitral flow and demarcating the early rapid filling phase and inflow deceleration phase. These phases are succeeded by diastasis, characterized by minimal pressure differentials. During LA contraction, LAP once again surpasses LVP, and an A wave appears. E: mitral peak velocity of early filling; A: mitral peak velocity of late filling.

Active relaxation and restoring forces rapidly reduce LV pressure (LVP) during isovolumetric relaxation [9]. Whereas passive relaxation is predominant during isovolumetric relaxation, active relaxation is the initiating event and also participates therein. During isovolumetric relaxation, both the aortic valve and the mitral valve remain closed, and LVP rapidly declines until it equals the left atrial pressure (i.e., LVP = LAP) (Figure 2), but the lengthening of myocytes and the hermetically sealed ventricular chamber jointly generate potential energy for sucking the blood from the left atrium (LA) to the LV apex.

As the SR takes up more calcium ions, the uncoupling between actin and myosin filaments increases, causing myosin heads to detach from actin binding sites. This allows the myofilaments to slide back into a relaxed state, in which actin and myosin filaments are no longer engaged in cross-bridge cycling. This disengagement facilitates muscle relaxation, enabling the cardiac muscle fibers to elongate. This process contributes to the reduction in LVP, which eventually falls below left atrial pressure (LVP < LAP) (Figure 2). This atrioventricular pressure gradient, which pulls blood toward the LV apex, can be considered a measure of LV suction and plays a crucial role in early LV filling (i.e., early rapid filling).

According to the simplified Bernoulli equation, the E wave, which represents the early diastolic flow velocity across the mitral valve, is determined by the pressure gradient between LA and LV. This relationship is expressed as ΔP = 4 V^2^, where ΔP is the pressure difference (LAP − LVP) and V is the flow velocity indicated by the E wave (in m/s). Consequently, the E wave velocity directly reflects the magnitude of this pressure gradient during early diastole. This gradient is influenced by changes in the rate of LV relaxation and filling pressure.

**Figure 3:**
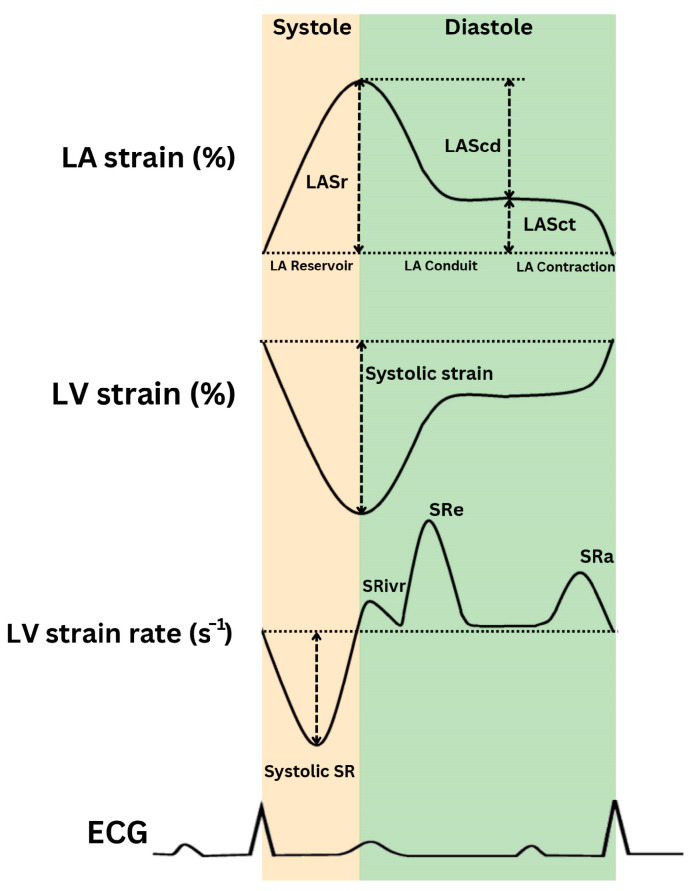
Schematic representation of left ventricular (LV) strain, strain rate, and left atrial (LA) strain (LAS). SR_ivr_: strain rate during isovolumic relaxation; SR_e_: strain rate during early diastole; SRa: diastolic peak longitudinal strain rate; ECG: electrocardiogram.

The LV untwisting rate (derived from LV short-axis views) and e′ (the peak early diastolic mitral annular velocity) are used to evaluate the LV early diastolic recoil capacity [6,10]. Additionally, e′, the LV longitudinal strain rate during isovolumic relaxation (SR_ivr_), and the LV strain rate during early diastole (SR_e_) are significantly associated with LV active relaxation [11] (Figure 3). Advancements in LV diastolic function assessment by strain and strain rate, derived from 2D-speckle-tracking echocardiography (STE), are discussed in Section 5.1.

With LV rapid filling, the pressure gradient between the LA and the LV apex decreases and briefly reverses (inflow deceleration). The reversed pressure gradient at the mitral valve slows and eventually halts the blood flow into the LV, marking the end of the rapid filling phase during early diastole (Figure 2). The duration of inflow deceleration (i.e., deceleration time [DT]), and A wave (late diastolic filling flow, as well as atrial contraction) velocity transit time, which is primarily influenced by the LV chamber’s functional stiffness, serve as noninvasive indicators of LV diastolic operational stiffness. During the mid-diastolic phase (i.e., diastasis), LAP and LVP equalize, and mitral flow nearly ceases. In late diastole, atrial contraction generates a second LA-to-LV pressure gradient, propelling blood into the LV. Then, as the LA relaxes, the LAP falls below the LVP, initiating mitral valve closure. In short, LV diastolic function is characterized by early diastolic recoil, LV relaxation, and chamber stiffness—all of which, in turn, determine the LV filling pressure (Table 1).

As the preceding discussion shows, the contraction and relaxation functions of the heart are interdependent. A stronger systolic contraction results in more significant recoil, thereby increasing potential energy during diastole. Additionally, when more calcium is actively taken up and stored in SR by sarcoendoplasmic reticulum calcium (SERCA), the SR subsequently releases more calcium through the ryanodine receptors (RyR, calcium-induced calcium release channels) during the systolic phase of the cardiac cycle (Figure 1), thereby enhancing myocardial contractility [13,14]. In cases of HF with preserved ejection fraction (HFpEF), although LV ejection fraction (LVEF) is in the normal range, the LV’s systolic performance is not quite normal [15], as indicated by a reduced LV twist during exercise [16]. The contraction and relaxation functions of the heart are tightly coupled, yet each function is also influenced by independent factors. Impaired LV relaxation, often an early sign of myocardial dysfunction, highlights the importance of both systolic and diastolic performance in maintaining overall cardiac health.

### Interpreting e′ and E/e′ in Diastolic Dynamics

The movement of the LV apex remains minimal throughout the cardiac cycle, making septal or lateral mitral annular motion a reliable proxy for longitudinal LV contraction and relaxation [17]. e′ coincides with the mitral E wave, indicating symmetrical LV expansion during early diastole as blood moves swiftly toward the LV apex, driven by a gradually increasing pressure gradient between the LV and LA. e′ is highly feasible, reproducible, and consistently associated with cardiovascular outcomes. It is influenced by three independent factors:

1. Restoring forces: The forces resulting from passive elastic recoil during LV relaxation, which cause the ventricle to return to its resting position. They reflect the mechanical and elastic properties of the myocardium, are generated by systolic contraction, and create a negative early diastolic pressure gradient that aids blood suction into the ventricle.
2. LV relaxation: The rate at which the active fiber force diminishes, signifying how quickly cardiac muscle cells return to their relaxed state after systole. LV relaxation reflects the heart’s ability to prepare actively for the next contraction cycle, crucial for facilitating diastole.
3. Lengthening load: The pressure in the LA at the mitral valve opening, which drives blood into the LV and elongates it. During mitral valve opening, the lengthening load and filling pressure typically align closely.

Restoring forces and LV relaxation characterize both passive relaxation during isovolumetric relaxation and active relaxation during rapid early diastolic filling. The E wave is directly proportional to the ratio of the filling pressure to the relaxation time constant (tau, τ), whereas e′ is inversely proportional to τ alone [18]. Thus, the E/e′ ratio and filling pressure are directly correlated [19], making E/e′ a practical and reproducible estimate of filling pressure that has been consistently correlated with pulmonary capillary wedge pressure (PCWP) across diverse patient populations in multiple research studies [20,21,22].

Sampling from at least two sites (between the tips of the mitral leaflets for E wave, and at the lateral and septal basal regions of the mitral annulus for e′) with adequate sample volumes is crucial. Notably, the correlation between E/e′ and LAP is strongest in patients with impaired LV systolic function but remains close in patients with preserved systolic function and varying loading conditions, such as those associated with aortic stenosis and exercise.

## 3. Abnormal LV Filling Patterns

### 3.1. LV Diastolic Dysfunction

Left ventricular diastolic dysfunction (LVDD) encompasses impaired relaxation, reduced restoring forces, early diastolic suction [23], and increased chamber stiffness [24,25], culminating in symptomatic HF due to elevated filling pressures at rest or with exertion [26]. Central to LVDD is the interaction between LVP and LAP. Elevated LV diastolic pressure is a hallmark of LVDD, contributing to blood accumulation in the LA and subsequent LAP elevation. Inadequate LV relaxation during diastole leads to a rise in LAP, which in turn impairs cardiac function and hemodynamics. The dynamic relationship between LVP and LAP is crucial for assessing LVDD severity, reflecting both heart filling status and LA workload. This comprehensive assessment is indispensable for diagnosing, treating, and evaluating cardiovascular conditions and overall cardiac function.

#### 3.1.1. Early Stages of LVDD

**Figure 4:**
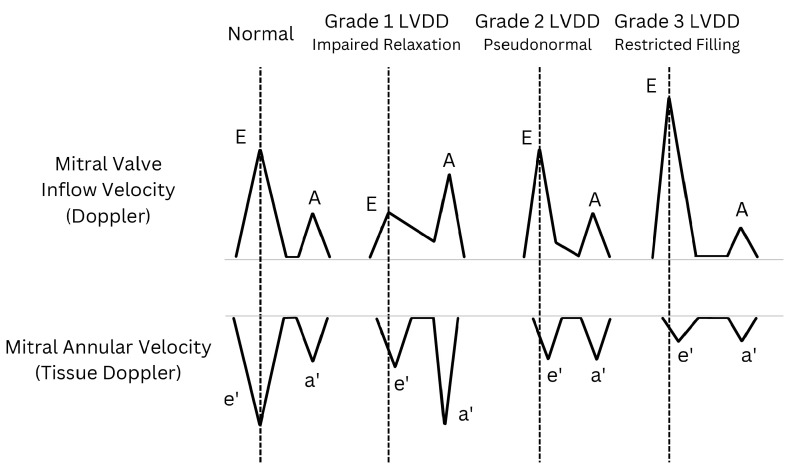
Diverse schematic patterns of left ventricular diastolic dysfunction (LVDD) illustrated through transmitral flow (**top**) and tissue Doppler at the level of the mitral annulus (**bottom**). As LVDD worsens, the peak value of e′ progressively decreases and is reached later in the cycle than the E wave peak (vertical dashed lines). E: mitral peak velocity of early filling; A: mitral peak velocity of late filling; e′: mitral annular peak velocity of early filling; a′: mitral annular peak velocity of late filling.

In mild LVDD cases in patients with relatively normal LAP [27], subtle changes manifest in the LV myocardium, namely minor reductions in relaxation and compliance. “Relaxation” refers to active myocardial relaxation, while “compliance” denotes passive recoil (Table 1). Reduced relaxation leads to a slight decrease in mitral annular velocity (e′). Delayed relaxation prolongs the E wave DT and may include a mid-diastolic peak (L wave) [23]. Conversely, decreased compliance causes a slight increase in early LV filling pressure, reducing the E wave. Atrial contraction compensates, resulting in an E/A ratio < 1, indicating an “impaired relaxation pattern” (i.e., grade 1 LVDD; Figure 4). Despite elevated LV end-diastolic pressure (LVEDP), mean LAP typically remains within the normal range in these patients, sustained by robust atrial contraction.

#### 3.1.2. Progression to Pseudonormal and Restricted Patterns

As LVDD advances, a pseudonormal mitral inflow pattern emerges (Figure 4). Elevated LAP re-establishes the early diastolic LA-to-LV pressure gradient despite increased diastolic LVP, potentially returning the E wave to its normal range. During this phase, delayed LV relaxation slows the e′ wave so that it occurs after the E wave, indicating asymmetrical LV expansion during diastole. With slow relaxation, the e′ wave becomes largely independent of LAP, contributing to reduced and delayed e′ velocity [28,29].

#### 3.1.3. Severe LVDD

In severe LVDD, characterized by significantly impaired relaxation and elevated LAP, a restricted filling pattern (i.e., grade 3 LVDD; Figure 4) becomes evident. The heightened E wave reflects impaired LV relaxation and sustained LAP elevation. Simultaneously, LAP rises early in diastole, surpassing the increase in LVP and further increasing the LA’s pressure gradient. This increase constrains LV filling, potentially causing blood stasis in the LA and impeding flow into the LV. The LA may enlarge over time to accommodate increased blood volume while maintaining elevated LAP. Severe LVDD can lead to a shortened E wave DT. This shortening of the E wave DT contributes to the reduction and delay of the e′ wave. As a result, the E/e′ ratio increases. Concurrently, peak late diastolic mitral annular velocity (a′) may decrease, and pulmonary venous systolic forward flow becomes slower than the diastolic flow.

#### 3.1.4. Clinical Significance

Pseudonormal (grade 2) and restricted (grade 3) LV filling patterns with elevated E/e′ ratios signify coexisting LVDD and elevated LAP, wherein blood is expelled from the LA rather than drawn into the LV [4,30,31]. E wave elevation primarily results from an increased LA-to-LV pressure gradient, whereas a reduced and delayed e′ wave indicates impaired LV filling. As LVDD progresses, LA remodeling secondary to increased filling pressures worsens symptoms, pulmonary vascular disease, and right ventricular dysfunction; reduces exercise capacity; and increases patients’ risk of adverse outcomes [32,33]. Some researchers advocate using reduced left atrial strain (LAS) [34,35] and increased LA stiffness (E/e′/LAS) [36] as diagnostic criteria for diastolic HF.

### 3.2. Echocardiography Parameters and Evaluation Algorithms for Diastolic Dysfunction

**Figure 5:**
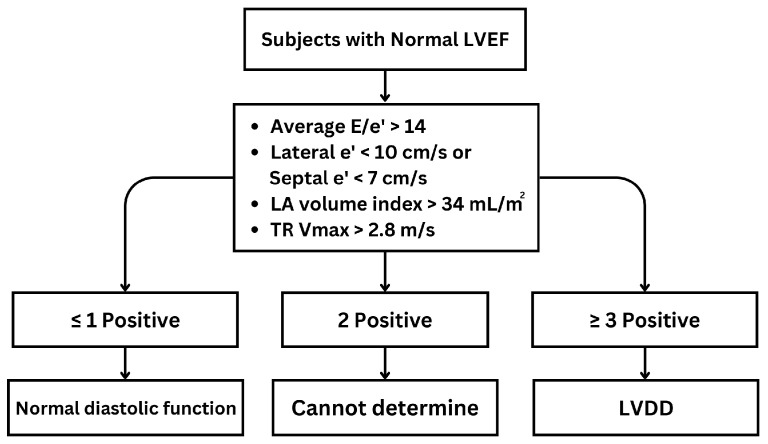
Algorithm A for diagnosis of LV diastolic dysfunction (LVDD) in subjects with normal LV ejection fraction (LVEF). E: mitral peak velocity of early filling; e′: mitral annular velocity of early filling by tissue Doppler; TR: tricuspid valve regurgitation; LA: left atrium; Vmax: maximum velocity.

**Figure 6:**
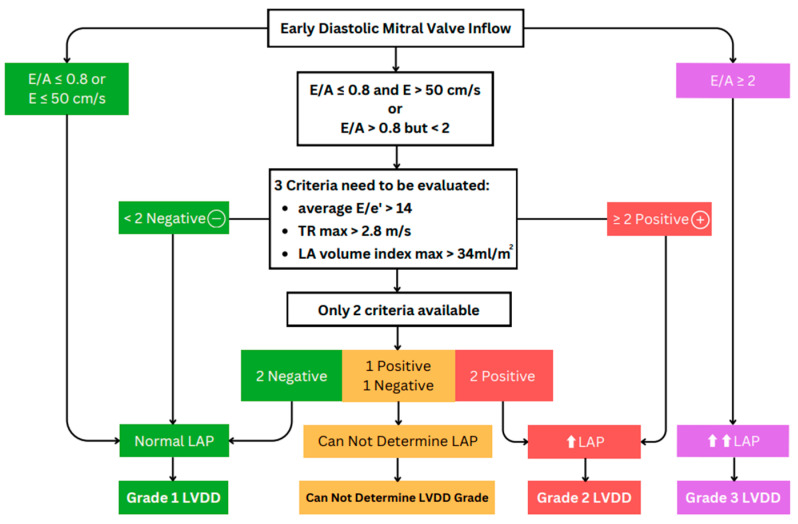
Algorithm B aims to estimate left ventricular (LV) pressure and grade LV diastolic dysfunction (LVDD) in patients with reduced LV ejection fraction (LVEF) and those with myocardial disease but normal LVEF. ⬆ and ⬆⬆ indicate a mild increase and a greater increase, respectively, in left atrial (LA) pressure. E/A: ratio of mitral peak velocities of early and late filling; e′: mitral annular velocity of early filling by tissue Doppler; TR max: peak velocity of tricuspid regurgitation; LA: left atrial; LAP: LA pressure.

The American Society of Echocardiography (ASE) guideline [23] introduces two distinct algorithms for assessing diastolic function. Algorithm A is aimed at patients with unknown diastolic function, and its primary purpose is to distinguish between normal and abnormal diastolic function (Figure 5). Algorithm B, conversely, is specifically designed for patients with known or suspected LVDD and focuses on estimating LV filling pressure and grading diastolic function. These two algorithms are valuable tools in the echocardiographic assessment of diastolic function across a spectrum of clinical scenarios, enabling clinicians to make informed diagnoses and treatment decisions. For algorithm A, abnormal diastolic function is defined as having ≥3 of the following abnormal parameters: annular e′ velocity with septal e′ < 7 cm/s, lateral e′ < 10 cm/s, average E/e′ ratio > 14, LA volume index (LAVI) > 34 mL/m^2^, and peak tricuspid regurgitation (TR) Vmax > 2.8 m/s. If only the lateral e′ or septal e′ velocity is available and clinically valid, a lateral E/e′ ratio > 13 or a septal E/e′ > 15 is considered abnormal. If there is a 50% discrepancy with two or four available variables, the findings are considered inconclusive for estimating LAP. Estimating LAP is not recommended if only one parameter provides a satisfactory signal [23]. Algorithm B is detailed in Figure 6.

LA volume is a crucial parameter for evaluating diastolic function and LV filling pressure [27], as it directly reflects LA dilation and remodeling. Nonetheless, measuring LA volume alone is insufficient for identifying LA dysfunction. LA deformation analysis, particularly LA reservoir strain, appears to be robust for detecting LA dysfunction [37,38]. In accordance with the ASE/European Association of Cardiovascular Imaging guidelines [23], LAS was evaluated as a marker of LVP alongside other echocardiographic parameters. This evaluation became especially crucial when other parameters, most notably TR velocity, were either missing or inadequately measured. The feasibility was high, as 99% of patients could be classified, and the accuracy was 82% [23].

PA systolic pressure (PASP) and mean wedge pressure are correlated. In patients without pulmonary disease, increased PASP is indicative of elevated LAP, after the exclusion of pulmonary hypertension types 1, 2, 4, and 5 [3]. PASP is calculated indirectly by using the Bernoulli principle from tricuspid regurgitation in systolic jet velocity (TR Vmax) [23,31]. A TR Vmax exceeding 2.8 m/s, corresponding to an estimated PASP of 32 mmHg, is associated with elevated LAP [23,39].

The differentiation between normal and abnormal diastolic function is complicated by the overlap between Doppler indices in healthy individuals and those with LVDD [23]. Individual parameters, including E/e′ and others discussed above, are no more than moderately associated with filling pressures and are insufficient when used independently [40]. Therefore, evaluating diastolic function requires using an integrated approach with three parameters [23]. If two of the three variables meet the cutoff values, this indicates an elevated LAP and grade II LVDD. If only one of the three available variables (Figure 6, third row, center box) meets the cutoff value, LAP is considered normal, indicating grade I LVDD. If there is a 50% discordance between two or four available variables, the findings are considered inconclusive for estimating LAP. Estimating LAP is not recommended if only one parameter provides a satisfactory signal.

The European Society of Cardiology (ESC) guidelines for the diagnosis and treatment of HF evolved between its 2016 and 2021 iterations. The 2021 guidelines [41] recommend using six objective signs of cardiac structural and functional abnormalities consistent with LV diastolic dysfunction or raised LV filling pressures: an increased LV mass index (≥95 g/m^2^ for women, ≥115 g/m^2^ for men), an enlarged LA (LAVI > 34 mL/m^2^), a resting E/e′ ratio > 9, a relative wall thickness > 0.42, PASP > 35 mmHg, and resting TR velocity > 2.8 m/s. The LA size and E/e′ criteria, plus mitral E velocity > 0.9 m/s and septal e′ velocity < 9 cm/s, are critical thresholds; values beyond these indicate greater risk of cardiovascular mortality.

The algorithm outlined above has significant limitations in that it does not apply to several specific cardiovascular diseases, including atrial fibrillation, noncardiac pulmonary hypertension, severe mitral regurgitation, mitral annular calcification, restrictive or hypertrophic cardiomyopathies, left bundle branch block, and paced rhythms [42].

## 4. Stress Echocardiography Testing in Diastolic Function Assessment

As discussed above, patients with LVDD may have a similar hemodynamic profile (in terms of cardiac output and filling pressure) at rest as healthy individuals who have normal diastolic function. The diastolic stress test uses exercise Doppler echocardiography (i.e., SE) to detect impaired LV diastolic functional reserve and the resulting increase in LV filling pressures [43,44,45,46]. It is a noninvasive hemodynamic test used to assess patients with unexplained dyspnea. It can also improve the diagnosis of HFpEF or diastolic HF. Frequently, symptoms of LVDD manifest only during exercise because it raises LV filling pressure [44]. The 2022 American College of Cardiology HF guidelines state that exercise SE evaluation of diastolic parameters can be helpful if the diagnosis remains uncertain after standard clinical assessment and resting diagnostic tests [47].

### 4.1. Stress Echocardiography during Relaxation in Healthy Individuals

Normal diastolic function enables the LV to adapt effectively to increased cardiac output during periods of stress or exertion. This adaptability is due to enhanced myocardial relaxation and more powerful early diastolic suction, neither of which significantly raises filling pressures. The E wave, representing early passive filling and relaxation rate, may increase slightly during stress due to elevated heart rate and increased cardiac output. Simultaneously, e′, reflecting the longitudinal rate of myocardial relaxation, increases proportionally during exercise. Faster myocardial relaxation indicates higher stress/exercise capacity. Consequently, the E/e′ ratio, which is an indicator of LV filling pressure, typically remains within the normal range [48,49,50] because both mitral inflow and annular velocities increase proportionately [51].

During exercise, the limited time available for diastolic LV filling due to tachycardia necessitates the acceleration of myocardial relaxation and the enhancement of LV suction to maintain or increase stroke volume while preserving normal filling pressure.

Various degrees of e′ elevation during exercise, reflecting longitudinal functional reserve, can be used as a parameter for assessing LV diastolic reserve during exertion [44,52]. Some studies test diastolic functional reserve to diagnose stress-induced LVDD, which is calculated as the product of Δe′ (the change in e′ from baseline to exercise) and baseline e′ (early diastolic mitral annular velocity at rest) [53]. Research has associated both exercise E/e′ and diastolic reserve with exercise capacity [54], particularly in patients with HFpEF [52,55]. The E/e′ ratio can also be utilized to estimate LAP or PCWP during both exercise and rest [56] (Table 2).

### 4.2. Stress Echocardiography in Patients with Left Ventricular Diastolic Dysfunction

In patients with LVDD, the pattern revealed by SE differs markedly from that of a healthy person. The E wave increases significantly to augment stroke volume, highlighting the challenges posed by impaired relaxation and elevated filling pressures. Conversely, e′ does not change as substantially as the E velocity in patients with abnormal myocardial relaxation [57], which is also reflected in a reduced diastolic functional reserve. This difference may be attributed to a pathological decline in the intrinsic relaxation capacity of the myocardium, affecting both active and passive relaxation. Consequently, even when stress increases the body’s demand for cardiac output, the heart of a patient with LVDD may not be able to augment myocardial relaxation to the necessary degree. This deficiency necessitates a higher filling pressure to maintain adequate blood filling and stroke volume. When E is elevated while e′ either increases slightly or remains relatively unchanged, the E/e′ ratio increases significantly (Table 2). This observation aligns with the previously mentioned greater increase in LVP during stress conditions.

In summary, SE testing offers valuable insights into diastolic function and filling pressures, distinctly differentiating between normal diastolic function and LVDD. This differentiation assists clinicians in evaluating cardiac performance under various conditions and in diagnosing LVDD. According to published studies, an E/e′ > 15 (using septal e′ velocity) can be used as a diagnostic criterion for stress-induced relaxation dysfunction [44].

## 5. Other Advanced Echocardiographic Techniques for Evaluating LV Diastolic Function

### 5.1. Strain Imaging

Strain, a measure of deformation, is a critical parameter in clinical cardiology. It is quantified as the percentage change in myocardial length between diastole and systole, thereby capturing the multifaceted spatial dynamics of cardiac contraction at both the global and regional levels [58]. Recent advancements in the field include the adoption of STE for assessing diastolic function intraoperatively. This novel technique, validated by numerous studies [59,60,61], offers a significant advantage by enabling comprehensive evaluation across the entire LV [62], unlike regional assessment methods. Another advantage of STE is its lack of angle dependency, a limitation commonly associated with tissue Doppler imaging. STE achieves this by acquiring images at a high frame rate (50–80/s) [63], allowing more accurate and comprehensive cardiac imaging. Further reinforcing the utility of STE, numerous studies show that both LV and LA strain, along with strain rate, are reliable predictors of LV diastolic function [35,60,64,65]. These findings underscore the potential of STE as a transformative tool in the evaluation and management of cardiac function, particularly diastolic performance. A recent review highlighted that resting global longitudinal strain (GLS) matches the efficacy of SE measurements in cardiac function assessment. Its advantages include semi-automation, cost-effectiveness, and remarkable reproducibility [66].

#### 5.1.1. LV Strain and Strain Rate

Evaluating LV strain rate is crucial for diagnosing LVDD, especially in HFpEF patients. Research confirms that reduced LV longitudinal strain is a hallmark of LVDD, emphasizing its clinical relevance [67]. Studies have linked LV diastolic strain and strain rate to the LV relaxation time constant [11]; 2D STE is emerging as a key technique for early detection [68]. Animal studies have revealed that SR_ivr_ shows inverse changes with saline infusion and increases with dobutamine infusion, which also results in higher −dP/dt and a shorter time constant (τ). Additionally, SR_e_ demonstrated a significant inverse SR_ivr_ [11]. Furthermore, longitudinal and radial SR_e_ have been correlated with the extent of replacement fibrosis [69]. The E/SR_ivr_ and E/SR_e_ ratios are positively associated with mean wedge pressure, with E/SR_ivr_ demonstrating a stronger correlation than E/SR_e_ [11,70]. E/SR_ivr_ has been shown to be more accurate than the E/e′ ratio in detecting elevated LV filling pressure, especially in cases of segmental dysfunction and in patients with normal LVEF [11]. Notably, a study correlated SRa with LVDD stage; an SRa < 0.68 s^−1^ indicated grade 2 or 3 LVDD with 80% sensitivity and 81% specificity [23]. The ratio of E to longitudinal strain (E/LS) has proven more effective than traditional measures like E/A and E/e′ in identifying elevated LV pressures, with an optimal cutoff of 680 cm/s achieving 72% sensitivity and 88% specificity [71]. Furthermore, studies demonstrate that strain rates, particularly during dobutamine stress testing, correlate with myocardial structure due to their association with interstitial fibrosis [60,61,64].

However, strain rates have limitations, including being affected by variability in image quality, a lack of standardization in diastolic strain rate measurements, the need for more training for accurate acquisition and analysis, and poorer accuracy in patients with tachycardia due to frame rate constraints [16].

#### 5.1.2. LA Strain and Strain Rate

In diastolic function, the LA anatomy and mechanics have an important role in maintaining LV function and preventing symptoms. Research on LAS has increased, proving it essential for assessing LV diastolic function and filling pressures. More recently, it has been hypothesized that LAS could serve as an even earlier marker for subclinical LV dysfunction and remodeling [72]. LAS measures LA deformation, with parameters like reservoir (LASr), conduit (LAScd), and contractile (LASct) functions highlighting the LA’s role in blood storage, passive transfer, and active contraction during the cardiac cycle. Advances in LAS measurement have enhanced LA function analysis [73], making LA stiffness (E/e′/LAS) and LAS itself key to identifying LA dysfunction [74] and early LVDD signs, even when traditional echocardiograms are normal [75].

In healthy individuals, stress conditions enhance both LASr and LAScd [44]. However, limited research has been conducted on the LA’s contractile strain under stress. Some studies have shown that under stress conditions, the LA’s conduit strain and contractile strain appear to merge [76].

LA longitudinal strain, being angle-independent, surpasses Doppler’s limitations by providing consistent LA deformation measures [77]. Singh et al. found that LASr varies significantly with LVDD severity, having a diagnostic precision of up to 95% and identifying LVEDP > 16 mmHg with 90% sensitivity and 82.9% specificity, thus outperforming LASct, LAScd, and E/e′ in diagnostic accuracy [78]. Abnormal LAS occurs more frequently than abnormal LAVI in LVDD, highlighting LAS’s sensitivity in detecting early LVDD [79]. Morris et al. showed that combining LAS with LAVI enhances LVDD identification significantly [34]. Other investigators found that LAS below 23% also indicates a higher risk of hospitalization for HF, independent of age, sex, and LAVI [80]; however, these investigators argued that LASr should be considered in diagnosing LVDD, but not as a stand-alone index. They found the ideal thresholds for distinguishing between normal and elevated LVP to be 18% for LASr and 8% for LASct when elevated PCWP was defined as >12 mmHg. Similarly, the thresholds were determined to be 16% for LASr and 6% for LASct when PCWP > 15 mmHg or LVEDP ≥ 16 mmHg was the criterion for elevated LVP. When a cutoff of <18% was used, the accuracy of LASr in differentiating between normal and elevated (>12 mmHg) filling pressure was 75% in the total study cohort, 81% in patients with LVEF < 50%, and 72% in patients with LVEF ≥ 50% [42,81].

Anish et al. emphasized stress LA strain’s diagnostic and prognostic relevance, showing LAScd’s crucial role in LV volume enhancement during exercise and underlining the value of LA size and function in HFpEF and broader cardiovascular disease management [82].

#### 5.1.3. Myocardial Work Analysis

Myocardial work (MW) assessed by noninvasive echocardiography is an innovative approach to evaluating systolic cardiac function. Because it incorporates both GLS and afterload, MW provides a more comprehensive assessment of myocardial performance than traditional strain measurements. This integration offers additional insights into cardiac function, addressing some of the limitations of strain analysis and potentially improving the accuracy of cardiac function evaluation [83,84]. Inputting blood pressure into the software generates an MW bullseye plot similar to that of GLS, along with a noninvasive LV pressure–strain loop (PSL) that depicts the relationship between changes in LV pressure and myocardial strain. This analysis calculates four key parameters [85]:

- Global work index (GWI, mmHg%): Represents the total work carried out by the LV from mitral valve closure to mitral valve opening.
- Global constructive work (GCW, mmHg%): Positive work performed by a segment in systole and negative work (segment lengthening) during isovolumic relaxation.
- Global wasted work (GWW, mmHg%): Negative work (segment lengthening) during systole and positive work (segment shortening) during isovolumic relaxation.
- Global work efficiency (GWE, %): Represents the efficiency of MW. Calculated as GCW/(GCW + GWW).

In a comparison of MW, LVEF, and MW combined with LVEF in patients with chronic heart failure, MW performed significantly better than LVEF in diagnosing early-stage HFpEF, achieving 88% accuracy, compared with 82% for LVEF alone. When combined with LVEF, MW increased classification accuracy to 98% [85]. An impaired GWI was observed in patients with cardiomyopathy despite preserved LVEF [86]. For patients with newly diagnosed HFpEF, GWW shows the highest diagnostic performance for predicting the risk of first hospitalization [87]. Recently, D’Andrea and colleagues conducted a larger study investigating MW indices in HFpEF patients. They found that GWW was higher at rest and during exertion in HFpEF patients than in controls, despite normal LVEF and GLS. This suggests a subclinical impairment of both systolic and diastolic function in these patients [78]. These findings align with our previous discussion that, due to calcium coupling mechanisms, diastolic dysfunction in HFpEF is often accompanied by varying degrees of contractility impairment.

However, there is currently no standardized protocol for measuring and interpreting MW, resulting in inconsistent results across studies and clinical settings. Moreover, MW calculations require multiple parameters and an optimal acoustic window from all echocardiographic views, which may not be attainable in every patient, potentially hampering reproducibility [88].

### 5.2. Integrating Artificial Intelligence

Although the most recent guidelines aimed to simplify the assessment of diastolic function, this evaluation remains intricate, relying on the integration of numerous clinical and echocardiographic variables [89]. Guideline-based algorithms require doctors to measure multiple metrics in multiple echocardiographic views, which requires substantial time and skill, so the quality of clinical reporting of diastolic function is difficult to guarantee [90].

The intricacies of assessing diastolic function have inspired a burgeoning interest in leveraging artificial intelligence (AI) to automate this process. New AI-based approaches have demonstrated significant potential in recent studies. Choi et al. [91] explored the diagnostic accuracy of a machine learning (ML) model in HFpEF, achieving an impressive 99.6% concordance with human specialists in diagnosing diastolic HF. The ML algorithm, incorporating LVEF, LAVI, and TR velocity, notably surpassed the conventional six-parameter assessment of diastolic dynamics. Omar et al. [92] employed STE measurements to develop an AI model to predict increased LV filling pressure, a critical LVDD parameter. Validation against invasively measured increases in PCWP showed the model’s robustness, with an area under the curve of 0.88, showcasing the potential for automated diastolic function assessment.

Efforts have also been made to enhance LVDD phenotyping for better outcome prediction. Pandey et al. employed ML to design a model that can identify patients with elevated LV filling pressure more accurately than the ASE’s 2016 diastolic guidelines grading system [93]. Additionally, Chiou et al. [94] developed a prescreening tool for diastolic HF that analyzes intra-beat dynamic changes in the LV and LA. By analyzing linear signals of LV and LA length, area, and volume waveforms, they identified novel intra-beat dynamic patterns that evaluated diastolic function with high accuracy, sensitivity, and specificity.

In the latest investigation, Chen et al. [95] introduced three AI-assisted methods for diastolic function assessment, achieving better diagnostic accuracy than human experts following guidelines. The models showed favorable results in evaluating and grading LV diastolic function. Notably, when Doppler variables were unavailable, the AI models could interpret 2D strain metrics or videos from a single view, suggesting significant potential for labor and cost savings, as well as workflow streamlining, in clinical LV diastolic function assessment.

## 6. Clinical Implications

Evaluating LVDD through echocardiography is crucial in cardiovascular care [96]. Assessing LV function goes beyond explaining symptoms, contributing importantly to predicting outcomes in cardiovascular patients [97]. Echocardiography not only detects issues early but also enables precise diagnosis, helping healthcare providers address cardiac abnormalities at their early stages and prevent HF and complications. Additionally, echocardiography provides valuable prognostic insights, enhancing the understanding of potential risks and informing long-term treatment planning [98]. Consistent echocardiographic follow-up is essential for monitoring disease progression and ensuring treatment effectiveness over time. Besides its diagnostic and therapeutic roles, echocardiography educates patients by visually documenting their condition, thus enhancing their comprehension and encouraging adherence to prescribed treatments and lifestyle changes. In summary, using echocardiography for LVDD assessment ensures a proactive approach to managing cardiovascular health.

### 6.1. Heart Failure with Preserved Ejection Fraction

Approximately 50% of HF cases are classified as HFpEF [41], which is associated with greater morbidity and mortality rates than patients without HF have [47]. HFpEF is associated with LVDD and, frequently, some level of LV longitudinal systolic dysfunction [99]. HFpEF can be hemodynamically defined as a clinical syndrome characterized by the heart’s inability to effectively pump blood without the need for elevated cardiac filling pressures [100]. According to the 2021 ESC guidelines [41], the diagnosis of HFpEF should encompass symptoms and signs of HF, LVEF > 50%, and objective evidence of cardiac structural or functional abnormalities indicative of LVDD or elevated LV filling pressures (discussed above), including elevated natriuretic peptide levels (Table 3). Consequently, establishing the diagnosis requires objective—chiefly echocardiographic—evidence of elevated filling pressures [41]. The proposed reference diagnostic standard relies on symptoms such as dyspnea, elevated natriuretic peptide levels, and an LVEF > 50%. However, these criteria lack specificity, necessitating a confirmatory test. Invasive right heart catheterization is considered the diagnostic gold standard [101]. Echocardiography can distinguish between HFpEF and HF with reduced LVEF (HFrEF) in patients with unexplained dyspnea and without any significant valvular disease [102]; this capability adds strategic depth to therapeutic interventions for LVDD, particularly when echocardiography reveals the specific etiological factors involved.

These parameters for assessing diastolic function were shown to predict mortality in three major HFpEF clinical trials [1,103,104]. Using existing guidelines alone to interpret resting echocardiographic data can only identify 34% to 60% of patients with invasively confirmed HFpEF [105]. A 2020 study [106] found that diagnostic performance was better with a multivariable model incorporating echocardiographic, clinical, and arterial function than with a single variable.

SE to diagnose HFpEF: In many patients with HFpEF, cardiac filling pressure is normal at rest but increases abnormally during exercise due to multiple cardiovascular reserve limitations [107,108,109], making resting echocardiographic parameters less sensitive for HFpEF diagnosis in these cases [44]. Therefore, recent interest has focused on SE diastolic testing (analyzing the E/e′ ratio and TR velocity during exercise) for the diagnosis of early HFpEF in cases of unexplained dyspnea and for a detailed assessment of exercise physiology for better HFpEF phenotyping. A positive diastolic stress test is characterized by the fulfillment of three conditions during exercise: average E/e′ >14 or septal E/e′ ratio > 15, peak TR velocity > 2.8 m/s, and septal e′ < 7 cm/s (or, if only lateral velocity is acquired, lateral e′ < 10 cm/s) at baseline [81]. Incorporating exercise/stress echocardiographic data with an E/e′ ratio > 14 increased sensitivity from 78% to 90% and, consequently, increased the negative predictive value. However, it decreased specificity to 71% [55]. Performing diastolic stress testing alongside standard resting echocardiography improves diagnostic sensitivity, especially in patients with suspected HFpEF with normal estimated LV filling pressure at rest [110]. In patients with GLS < 16–18% and suspected HFpEF, diastolic stress testing should be considered [81]. For patients who require stress testing, the most well-validated protocol is bicycle (in semi-supine position) exercise testing [45].

Other echocardiographic methods to diagnose HFpEF are detailed as follows: A study involving 3342 participants showed that using LV GLS assessment in adults with preserved LVEF, defined by a GLS of −15.9% or less, found an LVDD prevalence of 9.2% at baseline and 9.0% at follow-up [111]. When assessed by invasive methods, LV GLS < 16% has a sensitivity of 62% and a specificity of 56% for the diagnosis of HFpEF [109].

Enlarged LA is frequent in patients with HFpEF and is associated with elevated cardiovascular risk and LV filling pressure [112]. A recent study that included more than 300 patients showed that elevated LV filling pressure is reflected in reductions in LASr and LAScd [42]. Recently, another large multicenter study on multimodality imaging in HFpEF highlighted the clinical significance of LASr in detecting elevated LV filling pressure [82].

Additionally, a meta-analysis of four studies suggested a reasonable diagnostic accuracy for LAS: a specificity of 93% and a sensitivity of 77% [108,113,114,115]. Other studies have shown that LASr correlates more strongly with invasive LVP than with LAVI, and that LASr can detect LV diastolic alterations and elevated LV filling pressure even when LAVI is normal [34]. In a study of patients with normal LV systolic function [116], weak associations were observed between LASr or LASct and LVP. In contrast, high normal values for LASct strongly predicted normal LVP in patients with normal LVEF. This suggests a potential role for LA strain assessment in patients with normal LV function [42,117]. Moreover, LASr and LA compliance (LAS/E/e′) are useful in distinguishing HFpEF from noncardiac causes of dyspnea, demonstrating comparable or superior accuracy to that of the commonly used echocardiographic indices of diastolic function [113]. The most recent guidelines for the multimodal evaluation of HFpEF incorporate LASr as a component in the echocardiographic assessment of LV filling pressure [81].

### 6.2. Hypertensive Heart Disease

Among several risk factors, hypertension remains the leading cause of cardiovascular mortality [118]. Even individuals with prehypertension have detectably impaired cardiac relaxation [119]. Several studies emphasize that LVDD precedes systolic dysfunction, particularly in patients with cardiovascular risk factors like hyperlipidemia, diabetes, hypertension, obesity, and smoking habits [120]. The continued elevation of blood pressure levels contributes to LVDD through multiple mechanisms, including heightened afterload, myocardial ischemia, and the development of myocardial fibrosis [121]. Myocardial fibrosis is the primary factor in altering diastolic properties, impairing myocardial relaxation and thus disrupting normal LV diastolic filling [122]. LVDD is an independent predictor of cardiovascular outcomes in hypertensive populations [123,124].

Resting echocardiography to diagnose hypertensive cardiopathy: In patients with myocardial disease, even when LVEF is within the normal range, recommendations suggest applying algorithm B, which is used to assess LV filling pressures in patients with reduced LVEF [122]. A study by Zhou et al. found that individuals with high blood pressure had significantly lower e′ and e′/a′ values, along with a significantly higher E/e′ ratio, than non-hypertensive controls [125]. In a large-scale study with a total of 2500 patients with uncomplicated essential hypertension, an enlarged LA diameter was observed in more than 20% of the participants [126]; this enlargement, which can cause long-standing elevations in LV filling pressure and increased LA size and volume, were associated with poor long-term mortality and morbidity [127].

SE to diagnose hypertensive cardiopathy: SE reveals a spectrum of vulnerabilities in hypertensive patients, including LVDD, compromised cardiac and contractile reserve, coronary microcirculation dysfunction, and alterations in cardiac autonomic balance. SE has a superior sensitivity to that of electrocardiography and perfusion stress testing, yet has a similar sensitivity [128]. During peak stress, hypertensive patients have a higher A and a lower E/A ratio than they have in the absence of stress [129]. A diastolic stress test involving exercise [50] proved beneficial for excluding ischemia and establishing a correlation between dyspnea and indicators of elevated filling pressures, such as an elevated E/e′, and the change in a normal LV inflow pattern or a pattern of altered relaxation to a pseudonormalized pattern during exercise. An E/e′ ratio > 13–15 is considered abnormal.

Other echocardiographic methods to diagnose hypertensive cardiopathy are listed as follows: Significantly, LVDD is strongly correlated with LV longitudinal systolic dysfunction, and it might arise even before LV concentric geometry develops [130]. During the systolic phase, longitudinal shortening decreases while radial thickening is preserved, yet circumferential shortening increases to maintain cardiac output. In the diastolic phase, early diastolic strain rate decreases, particularly in the longitudinal direction, even in the absence of a significant elevation in LV filling pressure. In this context, strain appears more sensitive than both conventional echocardiography and Doppler tissue imaging in detecting a reduction in intrinsic myocardial contractility in hypertensive patients [120].

In a study involving normotensive controls and three groups of patients with different degrees of hypertension, segmental parameters exhibited apical–basal gradients, with the lowest values in the basal septal segments and the highest in apical segments. Only SRa remained consistent among segments, but it increased gradually with rising blood pressure [65]. SR_e_ decreased, particularly longitudinally, without a significant rise in LV filling pressure, showing that strain is more sensitive than conventional methods in detecting reduced myocardial contractility in hypertensive patients before LV hypertension [120]. The absolute values of LAS (S-reservoir and S-conduit) and strain rate (Sr-reservoir and Sr-conduit) were notably lower in patients with essential hypertension and LV hypertrophy [131]. In a 2022 study on hypertensive patients, LAS emerged as a robust predictor for LVDD with increased LAP. Among patients with LVDD, LAS that exceeded the cutoff of 24.27% was far more prevalent in patients with increased LAP (78.9%) than in those without it (15.4%), suggesting that LAS is a valuable, highly sensitive measure for assessment and potential integration into routine practice [132].

## 7. Future Perspectives

Diastolic function assessment through echocardiography holds immense promise, propelled by rapid advancements in technology and an enhanced understanding of cardiac physiology. AI and ML applications are on the cusp of reshaping the landscape, deploying algorithms to automate intricate measurements and deliver nuanced analyses. Ongoing studies are striving to harness AI’s potential to refine diagnostic criteria and elevate risk stratification, signifying a pivotal shift toward precision medicine in the realm of LVDD. Simultaneously, strain imaging provides more comprehensive insight into cardiac dynamics, potentially heightening sensitivity to detect subtle changes. Researchers are actively exploring the seamless integration of these advanced imaging modalities into algorithms for diastolic function assessment, thereby enriching our diagnostic arsenal. In the most recent studies, efforts have been made to integrate STE measures of LAS with ML to enhance the classification of LVDD [133].

In the inadequately explored domain of molecular imaging and biomarkers for LVDD, identifying novel markers could be the key to early detection and personalized management, aspects not covered in this review. Initiatives to unveil the molecular and cellular processes underlying LVDD could pave the way for targeted imaging agents and blood-based assays that clinicians could use to assess diastolic function at the molecular level. There may be a shift toward patient-centric approaches, integrating patient-reported outcomes and preferences into diagnostic strategies for a more personalized approach to care. Collaborative and multidisciplinary research initiatives are gaining momentum, addressing standardization challenges and translating research findings into clinical practice. This collective endeavor is poised to usher in a new era in cardiovascular medicine, wherein innovative technologies and collaborative endeavors converge to redefine the diagnosis and management of LVDD, ultimately enhancing patient outcomes.

## 8. Conclusions

Reduced myocardial relaxation is among the earliest indicators of LV mechanical dysfunction. Exploring diastolic dynamics with echocardiography is crucial for understanding and addressing a spectrum of myocardial conditions, including myocardial ischemia, hypertensive heart disease, hypertrophic cardiomyopathy, and HFpEF. Echocardiography, as the primary imaging modality for assessing LVDD, provides valuable insights into its hemodynamic impact and improves prognostic accuracy. From detecting subtle changes to guiding personalized treatments, this approach encompasses resting echocardiography, SE, and STE, as well as the application of AI and ML. Its multifaceted role significantly contributes to improving patient outcomes. To enhance accuracy in estimating LV filling pressure and grading LV diastolic function, the ASE guideline strongly recommends a comprehensive approach that integrates clinical data with echocardiographic findings [23].

Looking ahead, the future of echocardiography in diastolic function assessment is bright, with AI and ML poised to further refine diagnostic accuracy and enable personalized patient assessments. Research into molecular imaging and biomarkers is expected to unlock new possibilities for early detection and targeted management of LVDD. The integration of patient-reported outcomes and preferences into diagnostic strategies marks a shift toward more personalized care.

In conclusion, echocardiography remains an indispensable tool in the evaluation of diastolic function. The field is evolving rapidly, with technological advancements and collaborative research efforts driving significant improvements in cardiac diagnostics and therapy. This evolution is set to redefine the management of LVDD, ultimately enhancing patient outcomes and advancing the field of cardiovascular medicine.

## Figures and Tables

**Table 1:** Hemodynamic variables characterizing LV diastolic function in different diastolic phases and echocardiographic parameters.

Cardiac Phase	Affecting Factors	Parameter
Isovolumetric relaxation (LAP < LVP)	Active relaxation induced by SR uptake of Ca^2+^ (Initial) Elastic recoil after contraction (Primary)	IVRT untwisting rate SR_ivr_
Early rapid filling (LAP > LVP)	Active relaxation induced by SR uptake of Ca^2+^ (Primary) Elastic recoil after contraction	E wave peak e′ wave peak SR_e_
Inflow deceleration (LAP < LVP)	LV stiffness	E wave deceleration time
Diastasis (LAP = LVP)	LA stiffness Heart rate	Length of diastasis E/e′/LAS [12]
A wave upstroke (LAP > LVP)	LAP	A wave peak
A wave downstroke	LA stiffness	E/e′/LAS

LAP: left atrial pressure; LVP: left ventricular pressure; SR: sarcoplasmic reticulum; Ca^2+^: calcium; LVRT: left ventricular (isovolumetric) relaxation time; IVR: (left ventricular) isovolumetric relaxation; SR_ivr_: (left ventricular) strain rate during isovolumetric relaxation; SR_e_: (left ventricular) strain rate during early filling; E wave: mitral peak velocity of early filling; A wave: mitral peak velocity of late filling; e′ wave: mitral annular velocity of early filling by tissue Doppler; LV: left ventricular; LA: left atrial; LAS: left atrial strain.

**Table 2:** Comparative analysis of normal diastolic function and diastolic dysfunction in stress echocardiography.

	E Wave (Early Filling)	e′ Wave (Relaxation Rate)	E/e′ Ratio (Filling Pressure)
Normal diastolic function	↑	↑	N
Diastolic dysfunction	↑	Slight↑/N	↑↑

In healthy individuals under stress, cardiac output rises efficiently without a substantial increase in LVEDP, owing to enhanced myocardial relaxation. In contrast, patients with LVDD attain the necessary cardiac output only through an increase in LVEDP, because these patients lack a sufficient early suction mechanism for normal LV filling during early diastole. E wave: mitral peak velocity of early filling; e′ wave: mitral annular velocity of early filling by tissue Doppler; ↑: increase; ↑↑: greater increase; N: insignificant/negligible change.

**Table 3:** Objective evidence of cardiac structural, functional, and serological abnormalities consistent with LVDD/raised LVP.

Parameter	Threshold
LV mass index	≥95 g/m^2^ (female), ≥115 g/m^2^ (male)
Relative wall thickness	>0.42
LA volume index	>34 mL/m^2^ (sinus rhythm)
E/e′ ratio at rest	>9
NT-proBNP	>125 (sinus rhythm) or >365 (AF) pg/mL
BNP	>35 (sinus rhythm) or >105 (AF) pg/mL
PA systolic pressure	>35 mmHg
TR velocity at rest	>2.8 m/s

LV = left ventricular; LA = left atrial; NT-proBNP = N-terminal pro-B-type natriuretic peptide; AF = atrial fibrillation; BNP = B-type natriuretic peptide; PA = pulmonary artery; TR = tricuspid regurgitation.
